# Preface

**Published:** 1836

**Authors:** 


### PREFACE.

The tenth volume of the WESTERN JOURNAL OF THE MEDICAL AND PHYSICAL SCIENCES, is now finished; and it is no longer an open question, whether the Profession in the Valley of the Mississippi will support a periodical edited and published within its bordres. When this work was commenced 10 years ago, the editor regarded it as an experiment. It has proved a successful experiment, so far as the patronage of the work is concerned; but he has been disappointed in himself and many of his friends. Circumstances which he could not foresee, have constantly interfered with his writing for it to the extent he intended; and not a few of those on whom he relied for assistance, and whose talents and attainments qualified them to give it, have failed in their expected co-operation. Still the work, although in the hands of many successive publishers, has steadily advanced in public favor; and must have contributed *something*, towards the promotion of medical science in the region for which it was designed. In claiming this for it, the Editor claims nothing for himself, further than the humble merit of having made such selections from foreign periodicals, as were calculated to bring western readers acquainted with the progress of discovery in practical medicine and surgery elsewhere. For the young friends, who have at different times been associated with him in the editorial duties, he might claim more, for some of them have written liberally, and, as he hopes, with benefit to many of their readers.

It is no secret, that the Western Journal is in the interest of the medical department of the Cincinnati College; to which it will continue devoted, as long as, in the judgement of the Editor, the spirit and practice of that department, are compatible with the interests and dignity of the profession. Some of its professors, particularly Dr. Harrison and Dr. Gross, have several times inscribed themselves on the pages of the Journal, and they and their colleagues will continue, with increasing liberality, to make it the vehicle of the results of their observations, researches and experience. And as the institution to which they belong, has acquired a permanent organization, their aid will not be temporary or reluctantly given. The readers of the Journal will, therefore, in future, find that the establishment of the school has increased the actual value of this work. Moreover, with the general growth of the West and South, the number of writers must increase, and communications of merit cannot fail to become more and more numerous. In short, if the work could be sustained for the past ten years, no apprehensions need be felt for the future; as the whole circumstances of the country will be more favorable to the enterprize than they were in past times.

*Cincinnati, March* 31*st*, 1837.

